# The Association between Gestational Age and Risk for Long Term Ophthalmic Morbidities among Offspring Delivered in Different Preterm Subgroups

**DOI:** 10.3390/jcm11092562

**Published:** 2022-05-02

**Authors:** Erez Tsumi, Itai Hazan, Tamir Regev, Samuel Leeman, Chiya Barrett, Noa Fried Regev, Eyal Sheiner

**Affiliations:** 1 Department of Ophthalmology, Soroka University Medical Center, Ben-Gurion University of the Negev, Beer-Sheva 8410101, Israel; tamirre@clalit.org.il (T.R.); leemans@post.bgu.ac.il (S.L.); barat@post.bgu.ac.il (C.B.); 2 Joyce and Irving Goldman Medical School, Faculty of Health Sciences, Ben-Gurion University of the Negev, Beer-Sheva 8410501, Israel; itaihaz@post.bgu.ac.il; 3 Department of Emergency Medicine, Soroka University Medical Center, Ben-Gurion University of the Negev, Beer-Sheva 8410101, Israel; noafri@clalit.org.il; 4 Department of Obstetrics and Gynecology, Soroka University Medical Center, Ben-Gurion University of the Negev, Beer-Sheva 8410101, Israel

**Keywords:** ophthalmic morbidities, retinopathy of prematurity, gestational age, preterm delivery

## Abstract

Objective: To investigate whether there is a linear association between the degree of prematurity and the risk for long-term ophthalmic morbidity among preterm infants. Study design: A population-based, retrospective cohort study, which included all singleton deliveries occurring between 1991 and 2014 at a single tertiary medical center. All infants were divided into four groups according to gestational age categories: extremely preterm births, very preterm births, moderate to late preterm births and term deliveries (reference group). Hospitalizations of offspring up to 18 years of age involving ophthalmic morbidity were evaluated. Survival curves compared cumulative hospitalizations and regression models controlled for confounding variables. Results: During the study period, 243,363 deliveries met the inclusion criteria. Ophthalmic-related hospitalization rates were lower among children born at term (0.9%) as compared with extremely preterm (3.6%), very preterm (2%), and moderate to late preterm (1.4%) born offspring (*p* < 0.01; using the chi-square test for trends). The survival curve demonstrated significantly different hospitalization rates between the gestational ages (*p* < 0.001). The regression demonstrated an independent risk for ophthalmic morbidity among extremely preterm born offspring (adjusted hazard ratio 3.8, confidence interval 1.6–9.2, *p* < 0.01), as well as very preterm and moderate to late preterm (adjusted hazard ratio 2.2 and 1.5, respectively) as compared with term deliveries. Conclusions: The risk for long-term ophthalmic-related hospitalization of preterm offspring gradually decreases as the gestational age increases.

## 1. Introduction

The consequences of prematurity are well established in literature, causing long-term and potentially severe effects on pediatric and infantile morbidity and mortality [1,2]. With incomplete maturation, preterm infants are at a greater risk for developing a wide spectrum of medical complications, including hypothermia, jaundice, respiratory disorders [3], immunologic problems [4], neurodevelopmental problems [5,6,7], increased susceptibility to infections, hypoglycemia and feeding problems. In accordance with preterm newborns’ shorter intrauterine periods, ocular development in preterm infants is also subject to a number of abnormal influences, given the reduced time for proper growth and support in a unique environment [8]. Previous literature demonstrated that children who were born very preterm (28–32 weeks) are at a significantly higher risk for abnormal visual and neurological development, when compared to children born at full term [9]. These abnormalities include retinopathy of prematurity (ROP), strabismus, color vision deficits, visual field defects, decreased visual acuity and refractive error [10].

The World Health Organization (WHO) subcategorizes preterm births based on gestational age: extremely preterm (less than 28 weeks delivery), very preterm (28 to 32 weeks delivery), moderate to late preterm (32 to 37 weeks delivery) [11].

A recent study showed that long-term ophthalmic morbidities of offspring is significantly associated with early term delivery [12]. Early preterm-born offspring were found to have an independent risk for long-term ophthalmic morbidity (adjusted hazard ratio 2.51, confidence interval 1.91–3.29) as compared with full term offspring. In our study, we sought to investigate one step further to understand the relative risk for long-term ophthalmic morbidity between the different subcategories of preterm deliveries.

## 2. Materials and Methods

A retrospective cohort study of all singleton pregnancies in women who gave birth between the years 1991 and 2014 was conducted. Data was taken from the Soroka Univer-sity Medical Center (SUMC), the major tertiary hospital in the Negev region of Israel and the largest birth center in the country. The Negev region has continued to see increasing immigration since the 1990s. Therefore, the present study was based on non-selective population data. The study protocol was a received informed consent exemption by the SUMC institutional review board and was exempt from informed consent. The study population included two different ethnic groups: Jewish and Bedouins, who differ in their economic status, levels of education, and traditional beliefs [13]. However, prenatal care services are available to all Israeli citizens free of charge (covered by universal national health Insurance) [13]. Nevertheless, prenatal care services utilization is lower in Bedouin women as compared to Jewish women for a variety of social, cultural, and geographical access issues [14,15].

The primary exposure was defined as pre-term delivery (before 37 weeks). The control groups consisted of newborns born at later gestations. Gestational age was based on the best obstetric estimate determined by providers and used for clinical decision making. The standard criteria used involved consideration of the clinical history and earliest ultrasound scan. If the last menstrual period (LMP) was certain and consistent with the ultrasound, dating was based on the LMP. If the ultrasound was not consistent with the LMP, or the LMP was unknown, ultrasound data were used to determine gestational age.

Excluded from the study were fetuses with congenital malformations or chromosomal abnormalities, as well as perinatal mortality cases (intrauterine fetal death, intra-partum death, and post-partum death) and nonsingleton births. All offspring were divided into four groups according to their gestational age at delivery: extremely preterm birth: 24–28 gestational weeks, very preterm birth: 28–32 gestational weeks, moderate to late preterm birth: 32–37 gestational weeks and term deliveries: above 37 weeks.

The long-term outcomes assessed included all hospitalizations of offspring at SUMC until the age of 18 years that involved ophthalmic morbidity. The term “ophthalmic morbidity” included four categories: visual disturbances, retinopathy of prematurity, ocular infection and hospitalization. All diagnoses during hospitalization were predefined according to a set of ICD-9 procedures and diagnostic codes, detailed in Table A1.

Follow-up was terminated once any of the following occurred: after the first hospitalization, due to any of the predefined ophthalmic morbidities, any hospitalization resulting in death of the child, or when the child reached 18 years of age (calculated by date of birth).

Data was collected from two databases that were cross-linked and merged: the computerized hospitalization database of SUMC (“Demog-ICD9”), and the computerized perinatal database of the Obstetrics and Gynecology Department. The perinatal database consists of information recorded immediately following an obstetrician delivery. Experienced medical secretaries routinely reviewed the information prior to entering it into the database to ensure maximal completeness and accuracy. Coding was carried out after assessing medical prenatal care records, as well as routine hospital documents.

The SPSS package 23rd ed. (IBM/SPSS, Chicago, IL, USA) was used for statistical analysis. Categorical data is shown in counts and rates. Associations between the gestational age categories, background and outcome characteristics were assessed using the chi-square and ANOVA tests. In order to demonstrate the cumulative hospitalization incidences over time among the study groups, a Kaplan–Meier survival curve was used, and the log-rank test was used to assess the difference between the curves.

For the purposes of establishing an independent association between specific gestational age and the future incidence of ophthalmic-related hospitalizations of the offspring, a Cox regression model was constructed. The model was adjusted for confounding and clinically significant variables, including maternal age and diabetes (pre-gestational and gestational). All analyses were two-sided, and a *p*-value of ≤0.05 was statistically considered.

## 3. Results

A total of 243,363 deliveries were included in the study; 405 were between 24–28 gestational weeks (0.2%), 1084 deliveries were between 28–32 weeks (0.4%), 14,956 deliveries were between 32–37 weeks (6.1%) and 226,918 occurred at ≥37 gestational weeks (93.2%).

Table 1 summarizes maternal characteristics by delivery week category. Maternal age was similar in all delivery groups, with an overall average of 28.08 ± 6.3. Diabetes was more likely to occur in deliveries between 32–37 weeks (7.5%). Hypertensive disorders were more common in deliveries between 28–32 weeks (17.7%).

Table 2 summarizes pregnancy outcomes of all four groups. Mean birth weight was positively associated to gestational age; ranging from 855.1 ± 429.5 for 24–28 weeks of age to 3264 ± 446.6 among the mature group, while small for gestational age (SGA) as well as low birth weight (LBW( were most common in the former group (13.3% and 97.5% respectively). Cesarean deliveries were significantly more common among women in 28–32 weeks of gestation (44.8%, *p*-value < 0.01).

**Figure 1:**
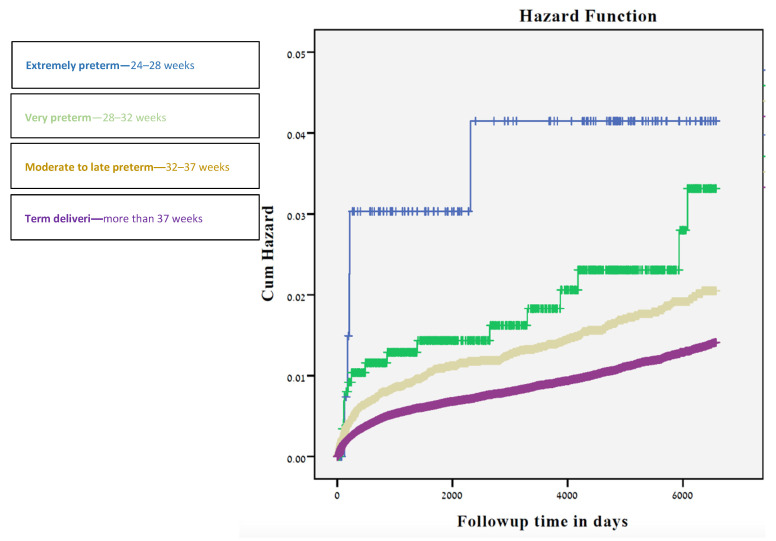
Kaplan-Meier survival curve demonstrating the cumulative incidence of. ophthalmological-related hospitalizations compared to delivery time in weeks.

The long-term ophthalmological morbidities based on hospitalizations is presented in Table 3. Rates of visual disturbances and retinopathy of prematurity (ROP) were significantly higher among the 24–28 gestational weeks group (0.7%, *p*-value < 0.01; 1.4%, *p*-value < 0.01 respectively), while ocular infection was highest among newborns in the 28–32 weeks of gestation (0.9%, *p*-value < 0.01). Total ophthalmic hospitalization rates were highest among the 24–28 gestational weeks group (3.6%, *p*-value < 0.01 using the chi-square test for trends), and gradually decreased with increasing gestational week (2% among 28–32 weeks, 1.4% among 32–37 weeks, and 0.9% among 37+ weeks). A decrease in the number of hospitalizations with gestational age was also documented while stratifying for ethnicity. Offspring born between 24–28 weeks had the highest cumulative incidence of hospitalizations (Figure 1) due to ophthalmological morbidity, followed by offspring born at 28-36 and those born at ≥37 gestational weeks (*p* < 0.001).

In the multivariate Cox regression model for offspring long-term risk of ophthalmic-related hospitalizations (Table 4), earlier deliveries were associated with higher risk for hospitalization, compared to the most mature group (24–28) weeks: HR = 3.8; CI95% 2.6–9.2; 28–32 weeks: HR = 2.2; CI95% 1.4–3.5; 32–37 weeks: HR = 1.5; CI95% 1.3–1.7).

## 4. Discussion

This large population-based cohort study demonstrates an increased risk for childhood and adolescence long-term ophthalmic morbidities as the gestational age becomes earlier among the pre-term deliveries. The risk remained significantly elevated among all the preterm groups while controlling for relevant maternal factors.

Numerous studies have identified an association between early gestational age at birth and the risk of poor health outcomes, based on incidence of morbidities, mortality and hospitalization rates [16,17]. A growing body of evidence has also shown ophthalmic morbidity among preterm infants. Kozeis et al. [18] found preterm infants to be associated with impairment of some aspects of visual function.

Although the pathophysiology is still not fully understood, complex multifactorial mechanisms have been suggested as the potential causes for ocular damage from preterm delivery. The suggested mechanisms include deficiencies in both innate and adaptive immunity [19], hypoxic-ischemic induced inflammation and cytokine injury, reperfusion injury, toxin-mediated injury, infection [20,21], and insufficient endogenous hormones (e.g., Cortisol & thyroxin), often exhibited as transient hypothyroxinemia of prematurity (THOP) [22].

Vast epidemiological studies conducted worldwide on ROP showed that despite geographical variability, the incidence of ROP was similar [14,15,16,17,18]. A study conducted by the Australian and New Zealand Neonatal Network (ANZNN) showed that the incidence of severe ROP was higher (34%) in infants born before 25 weeks gestation when compared to infants born at 25–26 weeks gestation (12.9%) [23]. Similar results have been found in North America, the UK and Indonesia [24,25,26].

Our findings revealed that the highest rate of ROP was observed in infants born at 24–28 weeks gestation (1.4%), with a declining rate for more advanced gestational age and reaching zero cases in those born after 32 weeks’ gestation. These results are consistent with previous studies examining the rate of ROP [27,28,29,30] and are likely to be explained by the fact that ROP is a disease of developing blood vessels, which are still considered underdeveloped at the beginning of the third trimester [31].

Additional long-term ophthalmological morbidities, such as visual disturbances, were examined in our study and showed the same pattern. The higher visual disturbances in preterm babies are a consequence of the higher retinopathy rate and other reasons e.g., osteopenia of prematurity [32]. Other types of vision disturbances include sub-categories, such as strabismus (both exotropia and esotropia), refractive errors, visual field deficits, color vision errors, astigmatism, cortical blindness and more. A recent study comparing strabismus in premature and term children found that the risk for strabismus in premature children was substantially higher than in full-term children (16.2% vs. 3.2%) [33]. Other ophthalmological morbidities related to prematurity, such as ROP, were linked to increased risk for strabismus as well [34].

Many factors contribute to the increased susceptibility of preterm infants to infections. Prematurity is associated with underdeveloped ocular surface defense mechanisms and has also been associated with neonatal conjunctivitis [35]. While previous studies showed an increase in the risk for general ocular infection among preterm newborns compared to term newborns [36], our analysis did not show a conclusive trend. Infants born between 24–28 weeks of gestation showed a lower proportion of ocular infections compared to those born during weeks 28–32 (0.7% compared to 0.9% respectively). This result could be explained by the smaller size of the preterm group and requires further analysis.

The key strength of our study was the use of a large cohort in a single medical center providing broad follow-up of children up to 18 years of age. This allowed for the investigation of substantially larger numbers of infants to be followed-up on than could be achieved by individual NICU follow-up studies, and simplified the monitoring of all infants for the purposes of comparison.

A major limitation of our study is its retrospective nature, as such studies may only suggest an association rather than causation. An additional limitation is the fact that information on other important risk factors, such as lifestyle, nutrition, perinatal treatment and other family factors was not available in the datasets. Two more challenges stemmed from the choice of ophthalmic-related hospitalizations as the present study’s endpoint: (1) Most of the ophthalmic morbidity is probably catered to in an outpatient setting (as discussed earlier). However, all significant ocular morbidity is routed to the SUMC, as it is the only tertiary hospital in the South of Israel (2) Offspring born earlier are more likely to be hospitalized in general, and therefore may be more frequently diagnosed with ophthalmic morbidities.

## 5. Conclusions

Our study was able to find a significant association between degree of prematurity and long-term ocular morbidities up to 18 years of age. A markedly increasing risk of severe adverse neonatal outcomes was observed as gestational age decreased. Further studies could raise awareness for early interventions, improve children’s health outcomes and decrease the burden on health care systems attributed to these diseases.

## Figures and Tables

**Table 1:** Maternal characteristics according to gestational age.

Maternal Characteristic	Extremely Preterm: 24–28 Weeks (*n* = 405)	Very Preterm: 28–32 Weeks (*n* = 1084)	Moderate to Late Preterm: 32–37 Weeks (*n* = 14,956)	Term Deliveries: more than 37 weeks (*n* = 22,6918)	*p*-Value
Ethnicity, *n* (%)					<0.01
Jewish	190 (46.9%)	463 (42.7%)	6883 (46%)	107,673 (47.5%)	
Bedouin	215 (53.1%)	621 (57.3%)	8073 (54%)	119,245 (52.5%)	
Maternal age, years, mean ± SD	28.44 ± 6.5	28.16 ± 6.3	28.14 ± 6.2	28.16 ± 5.7	<0.01
Diabetes ^1^, *n* (%)	5 (1.2%)	57 (5.3%)	1118 (7.5%)	10,973 (4.8%)	<0.01
Hypertensive disease ^2^, *n* (%)	40 (9.9%)	192 (17.7%)	1839 (12.3%)	10,169 (4.5%)	<0.01

^1^ Including pre gestational and gestational diabetes; ^2^ Including pre gestational, gestational hypertension and pre- eclampsia.

**Table 2:** Pregnancy outcomes for children (age < 18) by delivery week.

Pregnancy Outcome	Extremely Preterm: 24–28 Weeks (*n* = 405)	Very Preterm: 28–32 Weeks (*n* = 1084)	Moderate to Late Preterm: 32–37 Weeks (*n* = 14,676)	Term Deliveries: more than 37 Weeks (*n* = 226,917)	*p*-Value
Birthweight, gr mean ± SD	855.1 ± 429.5	1560.7 ± 623.4	2532.4 ± 506.3	3264.2 ± 446.6	<0.01
Small for gestational age ^1^, *n* (%)	54 (13.3%)	54 (5%)	607 (4.1%)	10,547 (4.6%)	<0.01
Low birth weight ^2^, *n* (%)	395 (97.5%)	992 (91.5%)	7030 (47%)	7805 (3.4%)	<0.01
Cesarean delivery, *n* (%)	139 (34.3%)	486 (44.8%)	4444 (29.7%)	27,908 (12.3%)	<0.01

^1^ Small for gestational age (SGA) < 5th percentile for gestational age; ^2^ Low birth weight (LBW) < 2500 g.

**Table 3:** Selected long-term ophthalmological morbidities for children (age < 18) by delivery week.

Ophthalmological Morbidity		Extremely Preterm: 24–28 Weeks (*n* = 138)	Very Preterm: 28–32 Weeks (*n* = 891)	Moderate to Late Preterm: 32–37 Weeks (*n* = 14,676)	Term Deliveries: more than 37 Weeks (*n* = 226,482)	*p*-Value
Visual disturbances, *n* (%)		1 (0.7%)	0 (0%)	36 (0.2%)	245 (0.1%)	<0.01
ROP, *n* (%)		2 (1.4%)	2 (0.2%)	0 (0%)	0 (0%)	<0.01
Ocular Infections, *n* (%)		1 (0.7%)	8 (0.9%)	123 (0.8%)	1336 (0.6%)	0.02
Total ophthalmic Hospitalization, *n* (%)	Bedouin	2 (1.4%)	12 (1.3%)	123 (0.8%)	1190 (0.5%)	<0.01
	Jewish	3 (2.2%)	6 (0.7%)	85 (0.6%)	907 (0.4%)	<0.01
	All	5 (3.6%)	18 (2%)	208 (1.4%)	2097 (0.9%)	<0.01

**Table 4:** Cox regression analysis of the association between week of birth and ophthalmic-related hospitalization of children (age < 18).

	Hazard Ratio	95% CI	*p*-Value
Extremely preterm: 24–28 weeks	3.8	1.6–9.2	<0.01
Very preterm: 28–32 weeks	2.2	1.4–3.5	<0.01
Moderate to late preterm: 32–37 weeks	1.5	1.3–1.7	<0.01
Term deliveries: more than 37 weeks	1 (Reference)		
Mother age at birth	0.9	0.98–0.99	0.02
Diabetes	1.0	0.8–1.2	0.6

## Data Availability

Unavailable according to the Helsinki protocol.

## Appendix A

**Table A1:** Supplement Data—Ophthalmic Diagnoses.

Group	Subgroup	Code	Diagnosis Description
*Ophthalmic*	Infectious inflammatory	370	KERATITIS
		3643	UNSPECIFIED IRIDOCYCLITIS
		3708	OTHER FORMS OF KERATITIS
		3709	UNSPECIFIED KERATITIS
		3720	ACUTE CONJUNCTIVITIS
		3723	OTHER AND UNSPECIFIED CONJUNCTIVITIS
		3729	UNSPECIFIED DISORDER OF CONJUNCTIVA
		3732	CHALAZION
		3736	PARASITIC INFESTATION OF EYELID
		3739	UNSPECIFIED INFLAMMATION OF EYELID
		36000	PURULENT ENDOPHTHALMITIS, UNSPECIFIED
		36011	SYMPATHETIC UVEITIS
		36012	PANUVEITIS
		36019	OTHER ENDOPHTHALMITIS
		36212	EXUDATIVE RETINOPATHY
		36320	CHORIORETINITIS, UNSPECIFIED
		36322	HARADA’S DISEASE
		36403	SECONDARY IRIDOCYCLITIS, INFECTIOUS
		36404	SECONDARY IRIDOCYCLITIS, NONINFECTIOUS
		36410	CHRONIC IRIDOCYCLITIS, UNSPECIFIED
		36424	VOGT-KOYANAGI SYNDROME
		37021	PUNCTATE KERATITIS
		37031	PHLYCTENULAR KERATOCONJUNCTIVITIS
		37040	KERATOCONJUNCTIVITIS, UNSPECIFIED
		37049	OTHER KERATOCONJUNCTIVITIS
		37055	CORNEAL ABSCESS
		37059	OTHER INTERSTITIAL AND DEEP KERATITIS
		37200	ACUTE CONJUNCTIVITIS, UNSPECIFIED
		37202	ACUTE FOLLICULAR CONJUNCTIVITIS
		37203	OTHER MUCOPURULENT CONJUNCTIVITIS
		37205	ACUTE ATOPIC CONJUNCTIVITIS
		37211	SIMPLE CHRONIC CONJUNCTIVITIS
		37213	VERNAL CONJUNCTIVITIS
		37214	OTHER CHRONIC ALLERGIC CONJUNCTIVITIS
		37220	BLEPHAROCONJUNCTIVITIS, UNSPECIFIED
		37230	CONJUNCTIVITIS, UNSPECIFIED
		37240	PTERYGIUM, UNSPECIFIED
		37261	GRANULOMA OF CONJUNCTIVA
		37300	BLEPHARITIS, UNSPECIFIED
		37311	HORDEOLUM EXTERNUM
		37312	HORDEOLUM INTERNUM
		37313	ABSCESS OF EYELID
		37400	ENTROPION, UNSPECIFIED
		37405	TRICHIASIS OF EYELID WITHOUT ENTROPION
		37500	DACRYOADENITIS, UNSPECIFIED
		37502	CHRONIC DACRYOADENITIS
		37530	DACRYOCYSTITIS, UNSPECIFIED
		37532	ACUTE DACRYOCYSTITIS
		37541	CHRONIC CANALICULITIS
		37542	CHRONIC DACRYOCYSTITIS
		37543	LACRIMAL MUCOCELE
		37600	ACUTE INFLAMMATION OF ORBIT, UNSPECIFIED
		37601	ORBITAL CELLULITIS
		37610	CHRONIC INFLAMMATION OF ORBIT, UNSPECIFIED
		37611	ORBITAL GRANULOMA
		37612	ORBITAL MYOSITIS
		37613	PARASITIC INFESTATION OF ORBIT
		37730	OPTIC NEURITIS, UNSPECIFIED
		37731	OPTIC PAPILLITIS
		37732	RETROBULBAR NEURITIS (ACUTE)
		37739	OTHER OPTIC NEURITIS
		37900	SCLERITIS, UNSPECIFIED
		37909	OTHER SCLERITIS
		376010	PERIORBITAL CELLULITIS
	Retinopathy of prematurity	36220	RETINOPATHY OF PREMATURITY, UNSPECIFIED
		36221	RETROLENTAL FIBROPLASIA
		36223	RETINOPATHY OF PREMATURITY, STAGE 1
		362211	RETINOPATHY OF PREMATURITY
	Visual disturbances	3670	HYPERMETROPIA
		3671	MYOPIA
		3672	ASTIGMATISM
		3682	DIPLOPIA
		3688	OTHER SPECIFIED VISUAL DISTURBANCES
		3689	UNSPECIFIED VISUAL DISTURBANCE
		3698	UNQUALIFIED VISUAL LOSS, ONE EYE
		3699	UNSPECIFIED VISUAL LOSS
		3780	ESOTROPIA
		36021	PROGRESSIVE HIGH (DEGENERATIVE) MYOPIA
		36720	ASTIGMATISM, UNSPECIFIED
		36731	ANISOMETROPIA
		36781	TRANSIENT REFRACTIVE CHANGE
		36800	AMBLYOPIA, UNSPECIFIED
		36801	STRABISMIC AMBLYOPIA
		36811	SUDDEN VISUAL LOSS
		36812	TRANSIENT VISUAL LOSS
		36840	VISUAL FIELD DEFECT, UNSPECIFIED
		36900	BLINDNESS OF BOTH EYES, IMPAIRMENT LEVEL NOT FURTHER SPECIFIED
		36960	BLINDNESS, ONE EYE, NOT OTHERWISE SPECIFIED
		36970	LOW VISION, ONE EYE, NOT OTHERWISE SPECIFIED
		37775	CORTICAL BLINDNESS
		37800	ESOTROPIA, UNSPECIFIED
		37801	MONOCULAR ESOTROPIA
		37805	ALTERNATING ESOTROPIA
		37810	EXOTROPIA, UNSPECIFIED
		37815	ALTERNATING EXOTROPIA
		37817	ALTERNATING EXOTROPIA WITH V PATTERN
		37820	INTERMITTENT HETEROTROPIA, UNSPECIFIED
		37821	INTERMITTENT ESOTROPIA, MONOCULAR
		37824	INTERMITTENT EXOTROPIA, ALTERNATING
		37830	HETEROTROPIA, UNSPECIFIED
		37831	HYPERTROPIA
		37832	HYPOTROPIA
		37835	ACCOMMODATIVE COMPONENT IN ESOTROPIA
		37881	PALSY OF CONJUGATE GAZE
		37885	ANOMALIES OF DIVERGENCE
	Not otherwise specified	3612	SEROUS RETINAL DETACHMENT
		3619	UNSPECIFIED RETINAL DETACHMENT
		3633	CHORIORETINAL SCARS
		3648	OTHER DISORDERS OF IRIS AND CILIARY BODY
		3651	OPEN-ANGLE GLAUCOMA
		3659	UNSPECIFIED GLAUCOMA
		3669	UNSPECIFIED CATARACT
		3769	UNSPECIFIED DISORDER OF ORBIT
		3770	PAPILLEDEMA
		3789	UNSPECIFIED DISORDER OF EYE MOVEMENTS
		3798	OTHER SPECIFIED DISORDERS OF EYE AND ADNEXA
		36020	DEGENERATIVE DISORDER OF GLOBE, UNSPECIFIED
		36023	SIDEROSIS OF GLOBE
		36030	HYPOTONY OF EYE, UNSPECIFIED
		36033	HYPOTONY ASSOCIATED WITH OTHER OCULAR DISORDERS
		36041	BLIND HYPOTENSIVE EYE
		36043	HEMOPHTHALMOS, EXCEPT CURRENT INJURY
		36044	LEUCOCORIA
		36100	RETINAL DETACHMENT WITH RETINAL DEFECT, UNSPECIFIED
		36103	RECENT RETINAL DETACHMENT, PARTIAL, WITH GIANT TEAR
		36104	RECENT RETINAL DETACHMENT, PARTIAL, WITH RETINAL DIALYSIS
		36105	RECENT RETINAL DETACHMENT, TOTAL OR SUBTOTAL
		36107	OLD RETINAL DETACHMENT, TOTAL OR SUBTOTAL
		36110	RETINOSCHISIS, UNSPECIFIED
		36133	MULTIPLE DEFECTS OF RETINA WITHOUT DETACHMENT
		36181	TRACTION DETACHMENT OF RETINA
		36202	PROLIFERATIVE DIABETIC RETINOPATHY
		36210	BACKGROUND RETINOPATHY, UNSPECIFIED
		36211	HYPERTENSIVE RETINOPATHY
		36216	RETINAL NEOVASCULARIZATION NOS
		36229	OTHER NONDIABETIC PROLIFERATIVE RETINOPATHY
		36231	CENTRAL RETINAL ARTERY OCCLUSION
		36234	TRANSIENT RETINAL ARTERIAL OCCLUSION
		36235	CENTRAL RETINAL VEIN OCCLUSION
		36240	RETINAL LAYER SEPARATION, UNSPECIFIED
		36250	MACULAR DEGENERATION (SENILE) OF RETINA, UNSPECIFIED
		36254	MACULAR CYST, HOLE, OR PSEUDOHOLE OF RETINA
		36256	MACULAR PUCKERING OF RETINA
		36263	LATTICE DEGENERATION OF RETINA
		36270	HEREDITARY RETINAL DYSTROPHY, UNSPECIFIED
		36273	VITREORETINAL DYSTROPHIES
		36274	PIGMENTARY RETINAL DYSTROPHY
		36281	RETINAL HEMORRHAGE
		36330	CHORIORETINAL SCAR, UNSPECIFIED
		36332	OTHER MACULAR SCARS OF RETINA
		36361	CHOROIDAL HEMORRHAGE, UNSPECIFIED
		36370	CHOROIDAL DETACHMENT, UNSPECIFIED
		36372	HEMORRHAGIC CHOROIDAL DETACHMENT
		36405	HYPOPYON
		36441	HYPHEMA OF IRIS AND CILIARY BODY
		36470	ADHESIONS OF IRIS, UNSPECIFIED
		36471	POSTERIOR SYNECHIAE OF IRIS
		36472	ANTERIOR SYNECHIAE OF IRIS
		36474	ADHESIONS AND DISRUPTIONS OF PUPILLARY MEMBRANES
		36475	PUPILLARY ABNORMALITIES
		36476	IRIDODIALYSIS
		36477	RECESSION OF CHAMBER ANGLE OF EYE
		36489	OTHER DISORDERS OF IRIS AND CILIARY BODY
		36500	PREGLAUCOMA, UNSPECIFIED
		36504	OCULAR HYPERTENSION
		36514	GLAUCOMA OF CHILDHOOD
		36520	PRIMARY ANGLE-CLOSURE GLAUCOMA, UNSPECIFIED
		36560	GLAUCOMA ASSOCIATED WITH UNSPECIFIED OCULAR DISORDER
		36600	NONSENILE CATARACT, UNSPECIFIED
		36610	SENILE CATARACT, UNSPECIFIED
		36612	INCIPIENT SENILE CATARACT
		36616	SENILE NUCLEAR SCLEROSIS
		36620	TRAUMATIC CATARACT, UNSPECIFIED
		36630	CATARACTA COMPLICATA, UNSPECIFIED
		36650	AFTER-CATARACT, UNSPECIFIED
		36813	VISUAL DISCOMFORT
		36815	OTHER VISUAL DISTORTIONS AND ENTOPTIC PHENOMENA
		36816	PSYCHOPHYSICAL VISUAL DISTURBANCES
		37000	CORNEAL ULCER, UNSPECIFIED
		37003	CENTRAL CORNEAL ULCER
		37004	HYPOPYON ULCER
		37100	CORNEAL OPACITY, UNSPECIFIED
		37105	PHTHISICAL CORNEA
		37120	CORNEAL EDEMA, UNSPECIFIED
		37123	BULLOUS KERATOPATHY
		37140	CORNEAL DEGENERATION, UNSPECIFIED
		37142	RECURRENT EROSION OF CORNEA
		37143	BAND-SHAPED KERATOPATHY
		37150	HEREDITARY CORNEAL DYSTROPHY, UNSPECIFIED
		37157	ENDOTHELIAL CORNEAL DYSTROPHY
		37158	OTHER POSTERIOR CORNEAL DYSTROPHIES
		37160	KERATOCONUS, UNSPECIFIED
		37162	KERATOCONUS, ACUTE HYDROPS
		37170	CORNEAL DEFORMITY, UNSPECIFIED
		37172	DESCEMETOCELE
		37263	SYMBLEPHARON
		37272	CONJUNCTIVAL HEMORRHAGE
		37273	CONJUNCTIVAL EDEMA
		37274	VASCULAR ABNORMALITIES OF CONJUNCTIVA
		37275	CONJUNCTIVAL CYSTS
		37289	OTHER DISORDERS OF CONJUNCTIVA
		37420	LAGOPHTHALMOS, UNSPECIFIED
		37430	PTOSIS OF EYELID, UNSPECIFIED
		37451	XANTHELASMA OF EYELID
		37482	EDEMA OF EYELID
		37484	CYSTS OF EYELIDS
		37489	OTHER DISORDERS OF EYELID
		37515	TEAR FILM INSUFFICIENCY, UNSPECIFIED
		37520	EPIPHORA, UNSPECIFIED AS TO CAUSE
		37521	EPIPHORA DUE TO EXCESS LACRIMATION
		37552	STENOSIS OF LACRIMAL PUNCTUM
		37553	STENOSIS OF LACRIMAL CANALICULI
		37554	STENOSIS OF LACRIMAL SAC
		37555	OBSTRUCTION OF NASOLACRIMAL DUCT, NEONATAL
		37556	STENOSIS OF NASOLACRIMAL DUCT, ACQUIRED
		37561	LACRIMAL FISTULA
		37630	EXOPHTHALMOS, UNSPECIFIED
		37633	ORBITAL EDEMA OR CONGESTION
		37641	HYPERTELORISM OF ORBIT
		37646	ENLARGEMENT OF ORBIT
		37651	ENOPHTHALMOS DUE TO ATROPHY OF ORBITAL TISSUE
		37681	ORBITAL CYSTS
		37689	OTHER ORBITAL DISORDERS
		37700	PAPILLEDEMA, UNSPECIFIED
		37703	PAPILLEDEMA ASSOCIATED WITH RETINAL DISORDER
		37710	OPTIC ATROPHY, UNSPECIFIED
		37721	DRUSEN OF OPTIC DISC
		37724	PSEUDOPAPILLEDEMA
		37741	ISCHEMIC OPTIC NEUROPATHY
		37749	OTHER DISORDERS OF OPTIC NERVE
		37852	THIRD OR OCULOMOTOR NERVE PALSY, TOTAL
		37853	FOURTH OR TROCHLEAR NERVE PALSY
		37854	SIXTH OR ABDUCENS NERVE PALSY
		37855	EXTERNAL OPHTHALMOPLEGIA
		37871	DUANE’S SYNDROME
		37887	OTHER DISSOCIATED DEVIATION OF EYE MOVEMENTS
		37923	VITREOUS HEMORRHAGE
		37924	OTHER VITREOUS OPACITIES
		37929	OTHER DISORDERS OF VITREOUS
		37931	APHAKIA
		37932	SUBLUXATION OF LENS
		37933	ANTERIOR DISLOCATION OF LENS
		37934	POSTERIOR DISLOCATION OF LENS
		37941	ANISOCORIA
		37942	MIOSIS (PERSISTENT), NOT DUE TO MIOTICS
		37943	MYDRIASIS (PERSISTENT), NOT DUE TO MYDRIATICS
		37950	NYSTAGMUS, UNSPECIFIED
		37951	CONGENITAL NYSTAGMUS
		37952	LATENT NYSTAGMUS
		37954	NYSTAGMUS ASSOCIATED WITH DISORDERS OF THE VESTIBULAR SYSTEM
		37955	DISSOCIATED NYSTAGMUS
		37956	OTHER FORMS OF NYSTAGMUS
		37959	OTHER IRREGULARITIES OF EYE MOVEMENTS
		37991	PAIN IN OR AROUND EYE
		37992	SWELLING OR MASS OF EYE
		37993	REDNESS OR DISCHARGE OF EYE
		37999	OTHER ILL-DEFINED DISORDERS OF EYEa
